# Giant planum sphenoidale meningioma: rationale for Dolenc’s step in the pterional approach

**DOI:** 10.3171/2025.10.FOCVID25172

**Published:** 2026-01-01

**Authors:** Akshohini Garg, Kuntal Kanti Das, Sudarsana Gogoi, Arun Srivastava

**Affiliations:** 1 Department of Neurosurgery, Sanjay Gandhi Post Graduate Institute of Medical Sciences, Lucknow; and; 2 Department of Pathology, Era’s Lucknow Medical College, Lucknow, Uttar Pradesh, India

**Keywords:** unilateral transcranial approach, optic canal unroofing, olfaction, anterior clinoidectomy

## Abstract

Large meningiomas of the midline anterior skull base do not respect anatomical boundaries. Therefore, a giant planum sphenoidale meningioma has the unique risk of simultaneously endangering the olfactory and visual functions. A pterional approach to such cases has several advantages, and the Dolenc’s modification allows a complete extradural anterior clinoidectomy, early release of compressed optic nerve, and exposure of basal portions of the internal carotid artery with a possible operative corridor expansion. This enhances tumor resection ergonomics with a more basal angle of attack while visualizing the anterior-to-posterior expanse of the entire anterior skull base.

The video can be found here: https://stream.cadmore.media/r10.3171/2025.10.FOCVID25172

## Transcript

In this video, the authors present "Giant planum sphenoidale meningioma: rationale for Dolenc’s step in the pterional approach."

### 0:27 Planum Sphenoidale Meningioma.

The planum sphenoidale meningiomas arise from the planum sphenoidale and are one of the three types of midline skull base meningiomas of the anterior skull base. When they are large, they can extend anteroposteriorly and can cause simultaneous visual and olfactory challenges. A pterional approach has several advantages, like early CSF release, avoidance of bifrontal retraction, preservation of olfaction, at least on one side, and the Dolenc’s step helps by early optic canal decompression, better identification of the compressed optic nerve, and better inferior-to-superior viewing angle. And all of this could be really advantageous in very large and challenging cases.

### 1:00 Case Capsule.

The case in point is a 27-year-old lady who presented with bifrontal headache for 3 years and painless progressive bilateral visual diminution, left worse than the right, for the last 1 year. On examination, there was no perception of light on the left side, whereas on the right side it was 6/18. Extraocular movements were full. Left-sided pupil was 4 mm and nonreacting, whereas the right-sided pupil was 3 mm and reacting well. On fundus, we could see bilateral primary optic atrophy. However, it was well developed and advanced on the left side.

### 1:28 Preoperative Imaging.

The preoperative MRI shows a T2-isointense large mass in the anterior midline skull base. On sagittal, we can see the anteroposterior extension. The tumor shows intense enhancement and possible hyperostosis of the planum sphenoidal area. We can also see tumor extension into the sella turcica. Therefore, we were dealing with a giant anterior skull base meningioma, epicentered over the planum sphenoidale and extending anteriorly to the olfactory fossa as well as posteriorly to the sellar region.

### 1:55 Operative Plan.

In this patient, we planned a left-sided pterional approach with Dolenc’s step, which involved zygoma lowering, to have an inferior-to-superior viewing angle; extradural clinoidectomy; and early optic canal unroofing. The rationale for our plan was it will allow an early CSF release and brain relaxation. Early optic nerve decompression can be achieved, which has good visual outcome. We can, at least possibly, save contralateral olfactory tract. We could better identify the compressed optic nerve, which is draped on the tumor and, sometimes, inadvertently damaged, if not detected early. And lastly, a better inferior-to-superior viewing angle, which will minimize brain handling during the removal of this tumor.

### 2:35 Position.

This was the patient’s position. The patient was supine with slight rotation of the head to the opposite side, and a frontotemporal incision was marked. In our practice, we prefer zygoma lowering over an orbital zygomatic approach. It was done in this patient.

### 2:48 Extradural Exposure and Clinoidectomy.

Following a frontotemporal craniotomy, we can see the frontal dura, temporal dura, and the orbital roof. The surgery started by a limited posterior orbitotomy. The meningo-orbital band was cut and the dural duplication was opened. We can see the V2 and the cavernous sinus in between V1 and V2 here, and glue was instilled inside by pressing on the sphenoparietal sinus. We can see the anterior clinoid process. The optic canal has been unroofed. The optic strut is drilled, and the clinoid process is delivered. The frontal scalp base is also lowered to have a subfrontal access. Basal dura is coagulated.

### 3:34 Tumor Resection.

Now we open the dura parallel to the scalp base—lower down. The advantage is we can preserve the brain. The optic nerve sheath was opened so that we could free the optic nerve early in the surgery before the tumor is handled. The arachnoid over the tumor is divided. We can see the posterior part of the ipsilateral olfactory tract. The posterior pole of the tumor is seen. Following arachnoid dissection, the tumor is debulked from the center. We can see the ipsilateral olfactory tract. Posteriorly, we have the optic nerve and the tumor medial to it. The tumor had actually compressed and displaced the optic nerve posteriorly.

### 4:25 Optic Nerve Preservation.

As we decompress the tumor, it becomes easier to identify the optic nerve now with the extradural part also in our vision. The arachnoid over the optic nerve, which is stretching onto the tumor, is divided, and gradually the optic nerve is mobilized away from the tumor. Preservation of the arachnoid is important.

To have a better view with minimal brain retraction, we additionally opened part of the sylvian fissure. We can see the optic nerve on the ipsilateral side. Further tumor decompression was done, and gradually the plane between the ipsilateral optic nerve and the tumor was developed. Anteriorly, we can see the tumor relationship with the basal frontal lobe. Again, the arachnoid over the tumor was sharply divided and gradually peeled away. The ipsilateral olfactory tract could not be preserved.

### 5:39 Preservation of Contralateral Olfactory Tract.

Here we can see the contralateral olfactory tract, which is located lateral to the tumor. Gently, the olfactory tract is being dissected away from the tumor capsule. It is important to preserve the arachnoid over the olfactory tract as much as possible.

More superiorly, the tumor is being rolled away from the brain, and we can appreciate the relationship of the anterior cerebral artery complex with the tumor.

### 6:11 Skeletonization of the ACAs.

Using gentle dissection, the artery was gradually freed. We can see that the anterior cerebral artery is very tightly adherent onto the tumor.

Using sharp dissection and tumor decompression, the artery is further freed, and we can see that the artery has been largely freed from the tumor. This is very important to prevent arterial spasm and infarct. As the tumor has been freed from the artery, we can gradually lift it off.

### 6:58 Contralateral Cranial Nerves’ Preservation.

 Here we have started to see part of the contralateral olfactory tract. The tumor is again debulked. This allows the tumor to be dissected away from the olfactory tract. It is very important to preserve at least one olfactory tract in this patient because the preoperative olfaction was intact. Posteriorly, we can see the compressed ipsilateral optic nerve. The tumor is being elevated from the sella, and in the interoptic space, we can see the optic chiasm, which is coming into our view. With gentle dissection, the tumor is lifted off the optic apparatus. We can see the contralateral optic nerve here.

Again, the optic chiasm and after sufficient separation of the tumor from the optic chiasm, the tumor is debulked again. We can see the dural base here.

### 8:15 Drilling of Bony Attachment.

Hyperostotic bone is seen, and we can now see the tumor, which was insinuating between the contralateral olfactory tract and the contralateral optic nerve. The tumor is gently separated while, at the same time, preserving the arachnoid over the contralateral olfactory tract. We can see the hyperostotic bone. The ipsilateral compressed optic nerve can be seen, the ICA below and lateral to it. Here we can see the amount of compression of the optic nerve. The hyperostotic bone was drilled. And this is the completed picture, where we can see the contralateral olfactory tract, ipsilateral optic nerve, and hemostasis was obtained.

### 9:03 Postoperative Clinical Outcome.

Postoperatively, the patient did not have any change in vision. However, olfaction on the right side remained preserved. At 3 months’ follow-up, the vision on the right side has improved to 6/12, whereas on the left side, there is mild improvement to hand movement perception. Although this is not significant yet, this is an improvement compared to the preoperative status. Importantly, there is preserved olfaction on the right side, and the patient remains functionally independent.

### 9:28 Postoperative Radiological Outcome.

Postoperative MRI, as shown here, shows near-total resection of the tumor. We can see that the frontal lobes are healthy and no residual tumor.

### 9:37 References.

These are the references.^1–6^ Thank you for watching this video.

## Disclosures

The authors report no conflict of interest concerning the materials or methods used in this study or the findings specified in this publication.

## Author Contributions

Primary surgeon: Das. Assistant surgeon: Garg. Editing and drafting the video and abstract: all authors. Critically revising the work: Das, Garg. Reviewed submitted version of the work: Das, Garg, Gogoi. Approved the final version of the work on behalf of all authors: Das. Supervision: Srivastava.

## Supplemental Information

### Patient Informed Consent

The necessary patient informed consent was obtained in this study.
